# Personals

**Published:** 1916-04

**Authors:** 


### Personals.

Dr. Thomas W. Wiley of McKinney has announced as candidate for Congress. He has been a resident of Texas for fifty years, and has practiced medicine in Collin county since he was a young man.

Dr. and Mrs. R. A. Foster of Nocona are visiting in San Antonio. The doctor has been in feeble health and it is hoped the trip will be beneficial.

Dr. J. C. Sellers of Spring has been suffering from painful bruises and cuts about the face and body, caused by his automobile overturning with him.

Dr. Geo. A. Taylor of Bettie is repoited to be dangerously ill with smallpox.

Dr. W. M. Griffis of Paris was overcome by gas from an instantaneous heater while taking a bath recently. Physicians were summoned. A pulmotor was brought and by its use his life was saved.

Dr. and Mrs. Denman of Robstown have gone to Chicago, where the doctor will attend lectures.

Dr. and Mrs. Levy of Beaumont are spending several weeks in Marlin for Mrs. Levy's health.

Dr. G. M. Stephens of Beeville attended the Rexal druggists' convention in San Antonio.

Dr. H. F. Leach of Weatherford left recently for New York City, where he will take a post-graduate course in surgery.

Dr. O. E. Nichols is in Chicago taking post-graduate work.

Dr. L. A. Brustad of San Antonio has gone to Corpus and other coast towns, where he will enjoy several weeks vacation from his practice.

Dr. J. W. McCarty of Owensboro, Kentucky, visited his brother in Beeville recently.

Dr. Cyrus Ray of Abilene and Miss Mary Montgomery of Fort Worth were married recently.

Dr. J. C. Grove of Gorman had a very painful operation performed recently, when a tumor was removed from his arm. Dr. Blackwell performed the operation, and his arm is healing nicely.

Dr. and Mrs. B. W. Mitchell of Buhlar, La., are guests in Orange, and it is understood they will locate there.

Dr. Roy Hughes of Gainesville and Miss Elsie Hay of Mineral Wells were married March 8th.

Dr. David Jackson Saunders and Miss Virginia Johnson of Fort Worth were married March 15th.

Dr. and Mrs. P. J. Pryor of Lott left recently for Tennessee, in response to information announcing the serious illness of Mrs. Pryor's father.

Dr. J. H. Hurt of Big Springs has just returned from a visit to Kentucky.

Dr. and Mrs. L. Penrod of Gonzales are going to Belmont to make their future home.

Dr. Norman and family of Sabinal have moved to Winters.

Dr. Z. M. Allen and wife of Sulphur Springs have returned from a five months' visit to the mountains of Arkansas.

Dr. Dubose of Oil City is back again at the Humble Pharmacy, and wishes to thank his friends who visited him during his recent sickness.

